# Atlases of cognition with large-scale human brain mapping

**DOI:** 10.1371/journal.pcbi.1006565

**Published:** 2018-11-29

**Authors:** Gaël Varoquaux, Yannick Schwartz, Russell A. Poldrack, Baptiste Gauthier, Danilo Bzdok, Jean-Baptiste Poline, Bertrand Thirion

**Affiliations:** 1 Parietal, Inria, Saclay, France; 2 Neurospin, CEA, Gif sur Yvette, France; 3 STIC department, Université Paris-Saclay, Saclay, France; 4 Psychology department, Stanford University, Stanford, CA 94305, USA; 5 Cognitive Neuroimaging Unit, INSERM, Gif sur Yvette, France; 6 JARA-BRAIN, Jülich-Aachen Research Alliance, Aachen, Germany; 7 Department of Psychiatry, Psychotherapy and Psychosomatics, RWTH Aachen University, 52072 Aachen, Germany; 8 Montreal neurological Institute and Hospital, McGill University, Montreal, Canada; Western University, CANADA

## Abstract

To map the neural substrate of mental function, cognitive neuroimaging relies on controlled psychological manipulations that engage brain systems associated with specific cognitive processes. In order to build comprehensive atlases of cognitive function in the brain, it must assemble maps for many different cognitive processes, which often evoke overlapping patterns of activation. Such data aggregation faces contrasting goals: on the one hand finding correspondences across vastly different cognitive experiments, while on the other hand precisely describing the function of any given brain region. Here we introduce a new analysis framework that tackles these difficulties and thereby enables the generation of brain atlases for cognitive function. The approach leverages ontologies of cognitive concepts and multi-label brain decoding to map the neural substrate of these concepts. We demonstrate the approach by building an atlas of functional brain organization based on 30 diverse functional neuroimaging studies, totaling 196 different experimental conditions. Unlike conventional brain mapping, this functional atlas supports robust *reverse inference*: predicting the mental processes from brain activity in the regions delineated by the atlas. To establish that this reverse inference is indeed governed by the corresponding concepts, and not idiosyncrasies of experimental designs, we show that it can accurately decode the cognitive concepts recruited in new tasks. These results demonstrate that aggregating independent task-fMRI studies can provide a more precise global atlas of selective associations between brain and cognition.

## Author summary

Cognitive neuroscience uses neuroimaging to identify brain systems engaged in specific cognitive tasks. However, linking unequivocally brain systems with cognitive functions is difficult: each task probes only a small number of facets of cognition, while brain systems are often engaged in many tasks. We develop a new approach to generate a functional atlas of cognition, demonstrating brain systems selectively associated with specific cognitive functions. This approach relies upon an ontology that defines specific cognitive functions and the relations between them, along with an analysis scheme tailored to this ontology. Using a database of thirty neuroimaging studies, we show that this approach provides a highly-specific atlas of mental functions, and that it can decode the mental processes engaged in new tasks.

## Introduction

A major challenge to reaching a global understanding of the functional organization of the human brain is that each neuroimaging experiment only probes a small number of cognitive processes. Cognitive neuroscience is faced with a profusion of findings relating specific psychological functions to brain activity. These are like a collection of anecdotes that the field must assemble into a comprehensive description of the neural basis of mental functions, akin to “playing twenty questions with nature” [1]. However, maps from individual studies are not easily assembled into a functional atlas. On the one hand, the brain recruits similar neural territories to solve very different cognitive problems. For instance, the intra-parietal sulcus is often studied in the context of spatial attention; however, it is also activated in response to mathematical processing [2], cognitive control [3], and social cognition and language processing [4]. On the other hand, aggregating brain responses across studies to refine descriptions of the function of brain regions faces two challenges: First, experiments are often quite disparate and each one is crafted to single out a specific psychological mechanism, often suppressing other mechanisms. Second, standard brain-mapping analyses enable conclusions on responses to tasks or stimuli, and not on the function of given brain regions.

Cognitive subtraction, via the opposition of carefully-crafted stimuli or tasks, is used to isolate differential responses to a cognitive effect. However, scaling this approach to many studies and cognitive effects leads to neural activity maps with little functional specificity, hard to assemble in an atlas of cognitive function. Indeed, any particular task recruits many mental processes; while it may sometimes be possible to cancel out all but one process across tasks (e.g. through the use of conjunction analysis [5]), it is not feasible to do this on a large scale. Furthermore, it can be difficult to eliminate all possible confounds between tasks and mental processes. An additional challenge to the selectivity of this approach is that, with sufficient statistical power, nearly all regions in the brain will respond in a statistically significant way to an experimental manipulation [6].

The standard approach to the analysis of functional brain images maps the response of brain regions to a known psychological manipulation [7]. However, this is most often not the question that we actually wish to answer. Rather, we want to understand the mapping between brain regions/networks and psychological functions (i.e. “what function does the fronto-parietal network implement?”). If we understood these mappings, then in theory we could predict the mental state of an individual based solely on patterns of activation; this is often referred to as *reverse inference* [8], because it reverses the usual pattern of inference from mental state to brain activation. Whereas informal reverse inference (e.g. based on a selective review of the literature) can be highly biased, it is increasingly common to use meta-analytic tools such as Neurosynth [9] to perform formal reverse inference analyses (also know as *decoding*). However, these inferences remain challenging to interpret due to the trade-off between breadth and specificity that is necessary to create a sufficiently large database (e.g. see discussion in [10, 11]).

The optimal basis for brain decoding would be a large database of task fMRI datasets spanning a broad range of mental functions. Previous work has demonstrated that it is possible to decode the task being performed by an individual, in a way that generalizes across individuals [12], but this does not provide insight into the specific cognitive functions being engaged, which is necessary if we wish to infer mental functions associated with novel tasks. The goal of decoding cognitive functions rather than tasks requires that the data are annotated using an ontology of cognitive functions [13–15], which can then become the target for decoding. Some recent work has used a similar approach in restricted domains, such as pain [16], and was able to isolate brain networks selective to physical pain. Extending this success to the entire scope of cognition requires modeling a broad range of experiments with sufficient annotations to serve as the basis for decoding.

To date, the construction of human functional brain atlases has primarily relied upon the combination of resting-state fMRI and coordinate-based meta-analyses. This approach is attractive because of the widespread availability of resting-state fMRI data (from which brain functional networks can be inferred through statistical approaches [17]), and the ability to link function to structure through the use of annotated coordinate-based data (such as those in the BrainMap [18] and Neurosynth [9] databases). This approach has identified a set of large-scale networks that are consistently related to specific sets of cognitive functions [19, 20], and provides decompositions of specific regions [21, 22]. However, resting-state analysis is limited in the set of functional states that it can identify [23], and meta-analytic databases are limited in the specificity of their annotation of task data, as well as in the quality of the data, given that it is reconstructed merely from activation coordinates reported in published papers.

**Fig 1:**
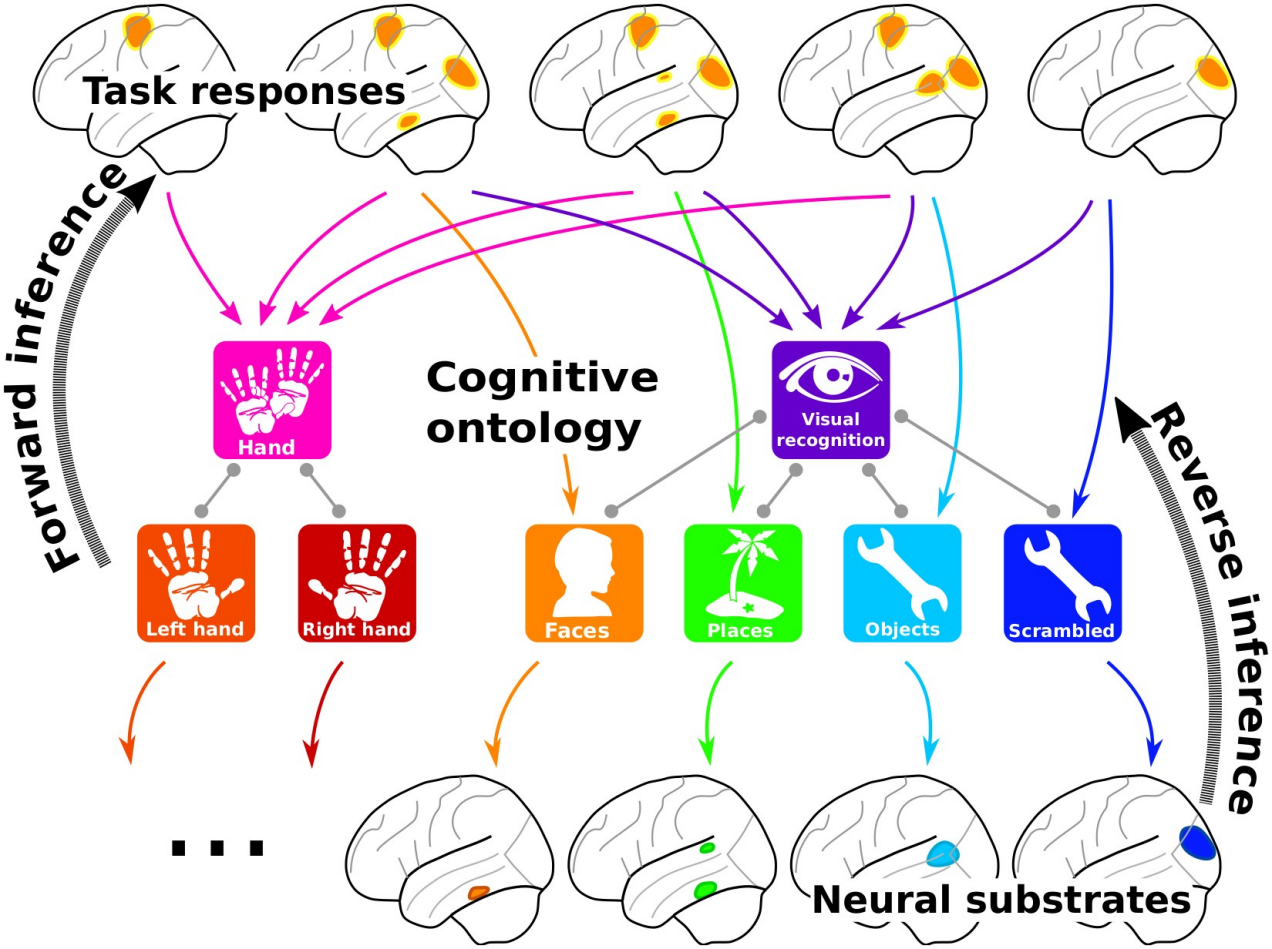
Brain mapping with a cognitive ontology. Our approach characterizes the task conditions that correspond to each brain image with terms from a cognitive ontology. *Forward inference* maps differences between brain responses for a given term and its neighbors in the ontology, i.e. closely related psychological notions. *Reverse inference* is achieved by predicting the terms associated with the task from brain activity. The figure depicts the analysis of visual object perception tasks with motor response. A forward inference captures brain responses in motor, primary visual and high-level visual areas. Reverse inference captures which regions or *neural substrate* are predictive of different terms, discarding common response to different tasks, here in the primary visual cortex.

A comprehensive functional brain atlas should link brain structures and cognitive functions in both forward and reverse inferences [7]. To build such a bilateral mapping, we introduce the concept of “ontology-based decoding,”, in which the targets of decoding are specific cognitive features annotated according to an ontology. This idea was already present in [9, 12, 24]; here we show how an ontology enables scaling it to many cognitive features, to increase breadth. In the present case, we use the Cognitive Paradigm Ontology (CogPO) [15], that provides a common vocabulary of concepts related to psychological tasks and their relationships (see S1 Text Distribution of terms in our database). Forward inference then relies on ontology-defined contrasts across experiments, while reverse inference is performed using an ontology-informed decoder to leverage this specific set of oppositions (see Fig 1 and methodological details). We apply these forward and reverse inferences to the individual activation maps of a large task-fMRI database: 30 studies, 837 subjects, 196 experimental conditions, and almost 7000 activation maps (see S1 Text Distribution of terms in our database). We use studies from different laboratories, that cover various cognitive domains such as language, vision, decision making, and arithmetics. We start from the raw data to produce statistical brain maps, as this enables homogeneous preprocessing and thorough quality control. The results of this approach demonstrate that it is possible to decode specific cognitive functions from brain activity, even if the subject is performing a task not included in the database.

## Materials and methods

### An ontology to describe cognitive neuroimaging studies

The main challenge to accumulate task fMRI is to account for the disparity in experimental paradigms. One solution is the use of cognitive ontologies that define terms describing the cognitive tasks at hand and enable to relate them. The choice of the ontology must meet two opposite goals: have a good coverage of the cognitive space, and document overlap between studies. In practice, each cognitive term describing mental processes must be expressed in several studies of our database to ensure the generalizability of our inference.

#### Terms

The cognitive ontologies currently being developed in the neuroimaging community follow two directions. The Cognitive Paradigm Ontology (CogPO) [15], which is derived from the BrainMap taxonomy [18], concentrates on the description of the experimental conditions that characterize an experimental paradigm. A taxonomy is a special case of ontology in which links between concepts are captured in categories: high-level concepts from categories that encompass lower-level concepts. In CogPO, experimental tasks are described via different categories that represent the stimuli, the expected responses, and the instructions given to the subjects, *e.g*., “stimulus modality”, “explicit stimulus”, “explicit response”. The CogPO terms are rather broad, but enable to find common task descriptors regardless of the original intent of the study. More tailored towards cognitive processes, the Cognitive Atlas [14] lists a large number of cognitive tasks and concepts, and increasingly links them together. We decide to mainly use terms from CogPO, and extend it where our database can benefit from more precise or high-level descriptions. Not all terms of CogPO in our database are present over multiple studies, and thus we only use a subset of CogPO. Similarly, with the limited number of studies in our database, there is only little overlap in high-level cognition. We added only the “language” label from the Cognitive Atlas.

It should be noted that the ontology does not have a full hierarchical structure, as *stimulus modality*, *explicit stimulus* and *explicit response* convey different level of information. Further work with growing databases will however need to add more and more terms. Finding a consistent structure underlying all these terms is a hard task.

#### Categories

Functional MRI experiments are carefully designed to balance conditions of interest with control conditions to cancel out effects related to the stimulation. As we do not want to ignore the designs, but rather leverage them in the context of a large-scale inference, we introduce an additional category level for our terms, that groups together terms –or conditions– that are typically contrasted in individual studies. These new categories strongly relate to the paradigm classes from BrainMap and the tasks from the Cognitive Atlas. The categories we choose are relevant to our database, and reflect the contrasts found in the studies. They nonetheless could be modified or extended further to test other hypotheses. This hierarchy of terms enables to co-analyze heterogeneous studies. S4 Table references the categories and associated terms used in this paper.

### Forward inference

Standard forward inference in functional neuroimaging uses the GLM (general linear model), which models brain responses as linear combinations of multiple effects. We use a *one-hot-encoding* of the concepts, *i.e*. we represent their presence in the tasks by a binary design matrix. We test for response induced by each concept with a second-level analysis using cross-studies contrasts.

To disentangle various experimental factors, brain mapping uses contrasts. Individual studies are crafted to isolate cognitive processes with control conditions, e.g. a face-recognition study would rely on a “face versus place” or a “face versus scrambled picture” contrast. To separate cognitive factors without a strong prior on control conditions, the alternative is to contrast a term against all related terms, e.g., “face versus place and scrambled picture”.

We use the categories of our ontology to define such contrasts in a systematic way for the wide array of cognitive concepts touched in our database. This approach yields groups of terms within the task categories, as described in Table 1: the task categories are used to define the conditions and their controls. Inside each group, we perform a GLM analysis with all the “one versus others” contrasts. We denote these *ontology contrasts*. Compared to a standard group analysis, the benefit of this GLM is that the control conditions for each effect studied span a much wider range of stimuli than typical studies.

**Table 1:** Contrasts used to characterize tasks effects in our database. We used CogPO categories for task-related description, and add necessary terms from Cognitive Atlas to describe higher-level cognitive aspect. Here we report only terms that were present in more than one study –aside from the “left foot”, which maps in the analysis as maps in “feet” task category, but not “right foot”. The task categories group terms typically used as conditions and their controls to test a hypothesis. The *stimulus modality* category stands for CogPO and task categories. Some terms do not belong to any task category and are referred to as such. The *arithmetics* task category spans across the *response modality* and *instructions* CogPO categories.

CogPO Categories	Task Categories	Terms	contrasts
Stimulus modality	-	visual	visual—auditory
		auditory	auditory—visual
Explicit stimulus	Sounds	human voice	human voice—sound
		sound	sound—human voice
	Retinotopy	vertical checkerboard	vertical checkerboard—horizontal checkerboard
		horizontal checkerboard	horizontal checkerboard—vertical checkerboard
	Object recognition	faces	faces—$\frac{1}{3}$(places + object + scramble)
		places	places—$\frac{1}{3}$(faces + object + scramble)
		objects	object—$\frac{1}{3}$(faces + places + scramble)
		scramble	scramble—$\frac{1}{3}$(faces + places + object)
	Symbol recognition	words	words—digits
		digits	digits—words
Response modality	Motor—hands	left hand	left hand—right hand
		right hand	right hand—left hand
	Motor—feet	left foot	left foot—right foot
		right foot	right foot—left foot
	Arithmetics	saccades	saccades
Instructions	Arithmetics	calculation	calculation
Cognitive Atlas term	No category	language	language

### Reverse inference

For reverse inference, we rely on large-scale decoding [12]. Prior work [12, 24] tackles this question using a multi-class predictive model, the targets of the classification being separate cognitive labels. Our formulation is different as our goal is to predict the presence or absence of a term, effectively inverting the inference of our forward model based on one-hot-encoding. This implies that each image is associated with more than a single label, which corresponds to multi-label classification in a decoding setting.

#### A hierarchical decoder

Linear models are widely used for decoding as they give good prediction and their parameters form brain maps. However, in a multi-label setting, they give rise to a profusion of separate one-versus-all (OvA) problems and cannot exploit the shared information between each label. We use a method based on stacked regressions [25]: two layers of linear models (logistic regressions) discriminating different cognitive terms. The first layer is tuned to specific oppositions between terms related in the ontology, while the second is tuned to predict which specific term is most relevant. This peculiar classifier architecture is tailored to the ontology that defines the structure of the targeted cognitive information. In the future, more complex cognitive ontologies may entail further refinements of the classifier.

#### First layer

First, we stack the decisions of the OvA classifiers, that capture specific activation patterns across all tasks. This allows to relate cognitive processes across independent cognitive disciplines. Second, we build one-versus-one (OvO) classifiers by opposing terms that belong to the same task category (see S4 Table). This enables to generalize the notion of contrasts and subtraction-logic that is implicit to the majority of fMRI experiments. Finally, we build classifiers predicting the actual task categories from S4 Table. It enables to build a hierarchical decoding framework, that combines the decisions of simpler problems, namely classifying the task categories, and more subtle, within-category problems: the OvO classifiers. There may be better choices of classifiers, but the final predictor weights them, and therefore mitigates the introduction of unnecessary or sub-optimal classifiers. We list in S2 Table all the classifiers that we use in the first level to learn the feature space capturing the ontology.

#### Second layer

**Fig 2:**
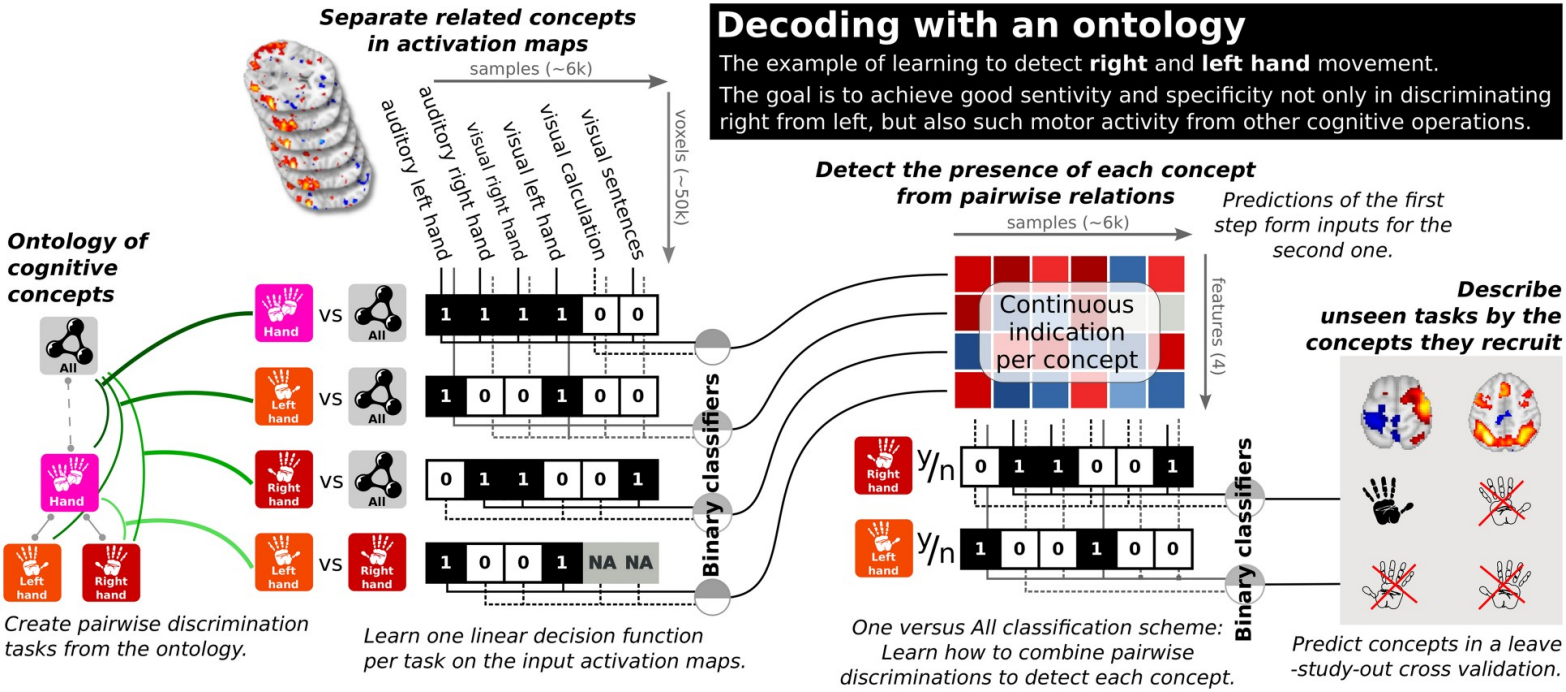
Ontology informed decoding. The hierarchical decoding procedure reduces the dimensionality by stacking the decision functions of several simple binary classifiers, which mimic study-level contrasts by opposing each term to matching ones. A second level of one-versus-all (OvA) classifiers predicts the presence of terms using the output of the first level. The first layer may be seen as capturing whether a given brain activity map looks more like face or place recognition, objects or scrambled images, visual or motor stimuli. The second layer combines this information to conclude on what cognitive terms best describe the given activity. Final linear classifiers may be recovered by combining the coefficients of the first and second level classifiers.

In a second layer, we learn the terms on the reduced representation with an OvA scheme, which also uses *ℓ*_1_-penalized logistic regression. The final output of this method is one linear classifier per term, that can be recovered by the linear combination of the coefficients of the base classifiers, with the coefficients of the final classifiers. The resulting ontology-informed decoder combines fined-grain information captured by opposing matching conditions in the first level with more universal decisions in the second level that outputs the presence or absence of a term. This combination is itself a linear classifier per label, and thus yields discriminant brain patterns for each term. Fig 2 summarizes this decoding procedure and S4 Text Reverse inference: Decoding with cognitive ontologies gives more specific details.

Such a two-step classification is important because binary classifiers opposing one term to another exhibit undesirable properties in rich output settings: for instance a binary classifier that would detect occurrences of *right hand* task would typically classify all *left hand* task occurrence as *right hand*, given that the negative class for this problem typically involved mostly non-manual tasks. Leveraging a two- instead of one-layer classification architecture creates the possibility to capture more subtle effects, a trick systematically used in recent deep learning models.

#### Cross validation

To evaluate the procedure, we perform the classification in randomized leave-3-study-out cross validation scheme. Cross-study prediction ensures that the representation of the cognitive labels generalizes across paradigms. We run 100 iterations of the cross validation to get a good estimate of the classifier’s performance.

## Results

### An atlas of areas linked to function

Using a database of 30 studies, we demonstrate that our approach captures a rich mapping of the brain, identifying networks with a specific link to cognitive concepts. Prediction of cognitive components in new paradigms validates this claim.

### Linking brain networks and cognitive concepts

**Fig 3:**
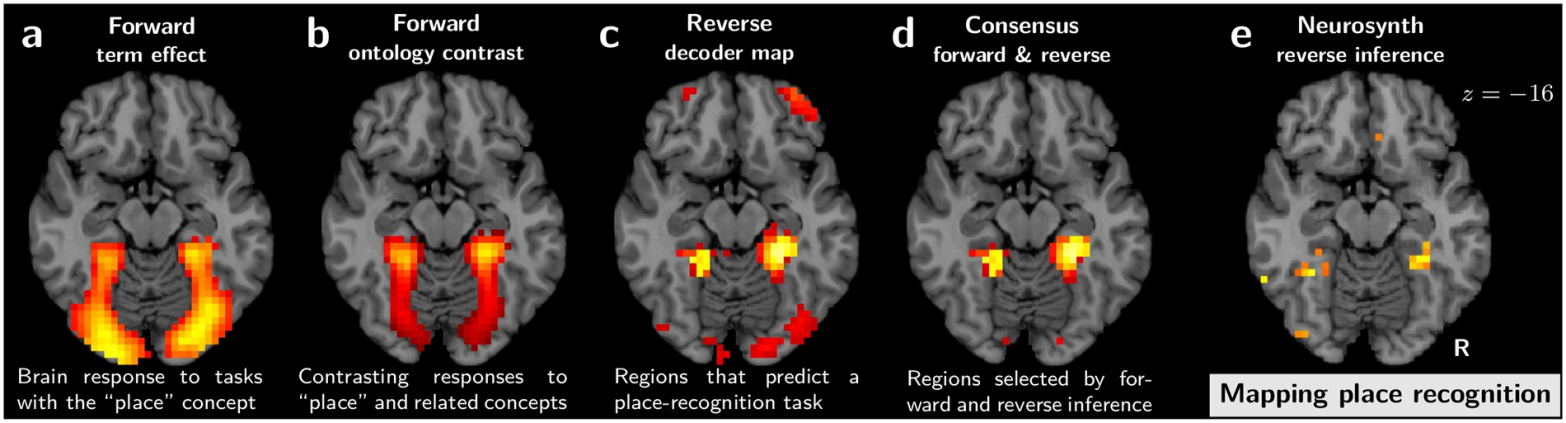
Maps for the different inference types. Left (**a**–**d**): maps of the different inferences on our database for the “place” concept. The consensus between reverse inference and forward inference based on contrasts defined from the ontology singles out the “parahippocampal place area” (PPA) for the “place” concept. Right (**d**): the NeuroSynth reverse-inference map for this concept. Reverse inference with Neurosynth also narrows well on the PPA, but is more noisy.

We combine forward and reverse inference to construct a one-to-one mapping between brain structures and cognitive concepts. Forward inference across studies requires adapting brain mapping analysis to leverage the ontology. Mapping the brain response to the presence of a concept in tasks selects unspecific regions, as it captures other related effects, *e.g*. selecting the primary visual cortex for any visual task (Fig 3). To obtain a more focal mapping, we remove these effects by opposing the concept of interest to related concepts in the ontology. Reverse inference narrows down to regions specific to the term. However, as we use a multivariate procedure, some of its variables may model sources of noise [26]. For instance, when using visual n-back tasks with a motor response to map the visual system, the motor response creates confounding signals. A multivariate procedure could use signal from regions that capture these confounds to subtract them from vision-specific activity, leading to better prediction. As such regions are not directly related to the task, they are well filtered with a standard GLM (General Linear Model) used in forward inference. For this reason, our final maps combine statistics from forward and reverse inference: functional regions are composed of voxels that are both recruited by the cognitive process of interest *and* predictive of this process; see S5 Text Consensus between forward and reverse inference for statistical arguments and [27] for more fundamental motivations regarding causal inference. Fig 3a–3d shows how the neural-activity patterns for the “places” label progressively narrow on the PPA with the different approaches. Thus we link each cognitive concept to a set of focal regions, resulting in a brain-wide functional atlas.

### Atlases with various mapping approaches

**Fig 4:**
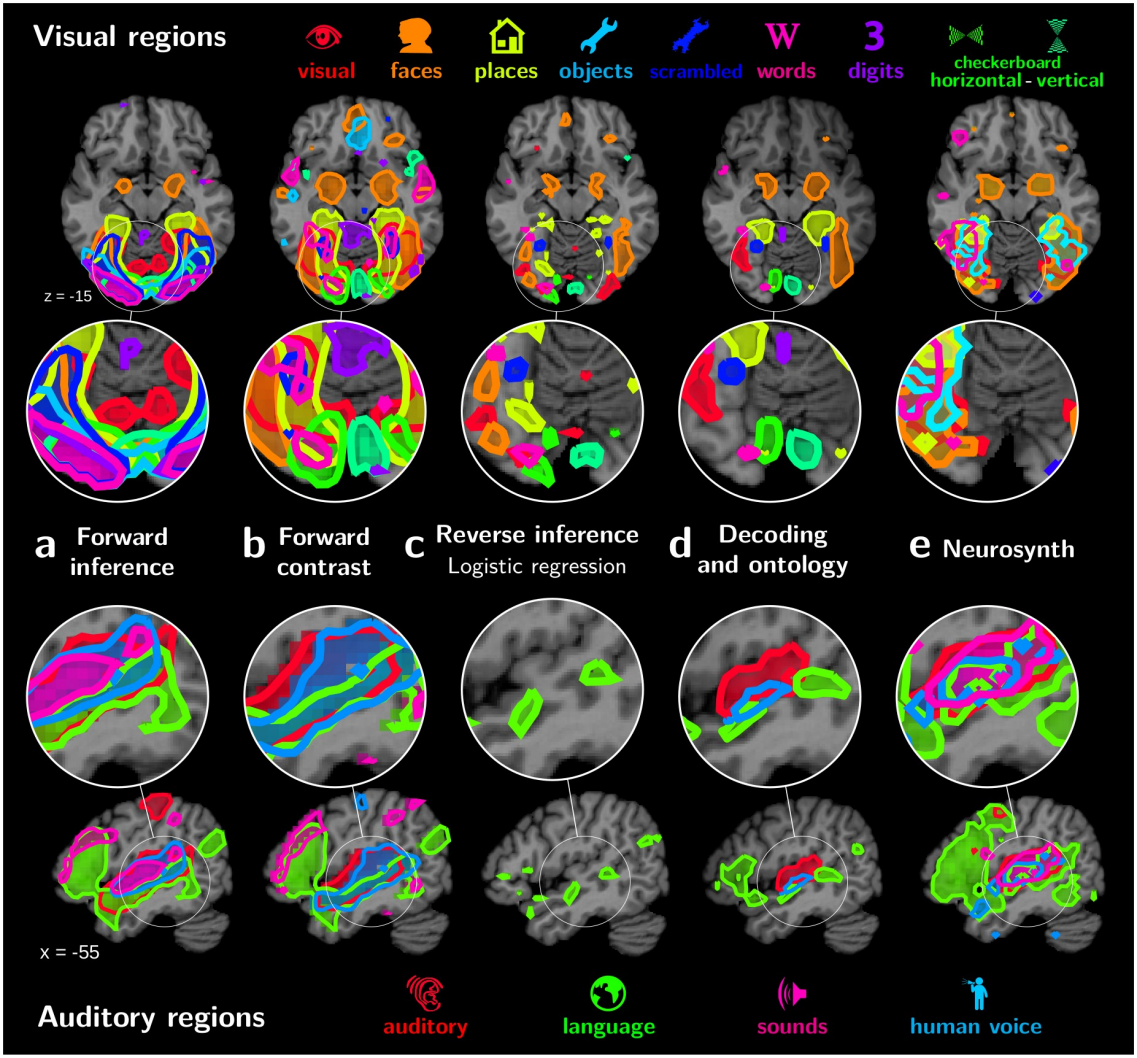
Different functional atlases. Regions outlined using different functional mapping approaches, from left to right: a. forward term mapping; b. forward inference with ontology contrasts (standard analysis); c. reverse inference with logistic regression; d. NeuroSynth reverse inference; and e. our approach, mapping with decoding and an ontology. The top part shows visual regions, and the lower one auditory regions in the left hemisphere. Forward term mapping outlines overlapping regions, as brain responses capture side effects such as the stimulus modality: for visual and auditory regions every cognitive term is represented in the corresponding primary cortex. Forward mapping using contrasts removes the overlap in primary regions, but a large overlap persists in mid-level regions, as control conditions are not well matched across studies. Standard reverse inference, specific to a term, creates overly sparse regions though with little overlap. Reverse inference with Neurosynth also displays large overlap in mid-level regions. Finally, ontology-based decoding maps recover known functional areas the visual and auditory cortices.

To build functional atlases, it is important to clearly identify the regions associated with different cognitive concepts. Fig 3e shows that reverse-inference meta-analysis with Neurosynth also associates the PPA with the “place” term, but the region is not as well delineated as with our approach. Fig 4 shows functional atlases of auditory and visual regions extracted with various mapping strategies. The relative position and overlap of the various maps is clearly visible. Forward-inference mapping of the effect of each term versus baseline on our database gives regions that strongly overlap (Fig 4a). Indeed, the maps are not functionally specific and are dominated by low-level visual mechanisms in the occipital cortex and language in the temporal cortex. Using contrasts helps decreasing this overlap (Fig 4b), and hence reveals some of the functional segregation of the visual system. However, as the stimuli are not perfectly balanced across experiments, contrasts also capture unspecific regions, such as responses in the lateral occipital cortex (LOC) for faces or places. Reverse inference with a logistic-regression decoder gives well separated regions, albeit small and scattered (Fig 4c). The ontology-informed approach identifies well-separated regions that are consistent with current knowledge of brain functional organization (Fig 4d). Finally, meta analysis with NeuroSynth separates maps related to the various terms better than forward analysis on our database of studies (Fig 4e). Yet some overlap remains, for instance in the LOC for maps related to visual concepts. In addition, the outline of regions is ragged, as the corresponding maps are noisy (Fig 3e), probably because they are reconstructed from peak coordinates. Note that overlaps across term-specific topographies are ultimately expected to remain, especially in associative cortices. In the following, we first discuss quantitative validation of the reverse-inference atlases, and then study in detail the atlas obtained with the ontology-informed approach.

### Decoding cognition validates the atlas

**Fig 5:**
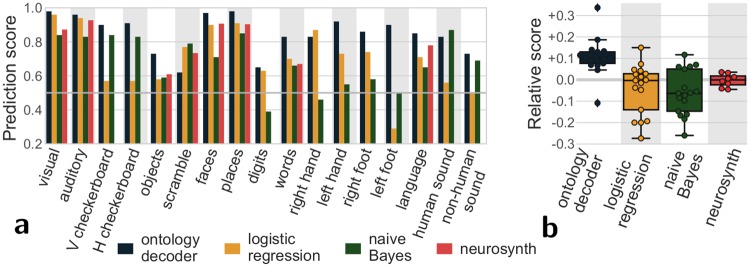
Prediction scores for different methods. Area under the ROC curve (1 is perfect prediction, while chance is at 0.5); **a** score for each term; **b** score relative to the average per term for each decoding approach. As the terms in NeuroSynth do not fully overlap with the terms used in our database, not every term has a prediction score with NeuroSynth. The ontology-informed decoder is almost always able to assign the right cognitive concepts to an unknown task and clearly out-performs standards decoders: logistic regression and naive Bayes classifier trained on our database. It also outperforms the NeuroSynth decoding based on meta-analysis.

Upon qualitative inspection, the regions extracted by our mapping approach provide a good functional segmentation of the brain. For an objective test of this atlas, we quantify how well these regions support reverse inference. For this, we use the ontology-informed decoder to predict cognitive concepts describing tasks in new paradigms and measure the quality of the prediction. This approach was tested using a cross-validation scheme in which 3 studies were held out of each training fold for subsequent testing. Fig 5 shows the corresponding scores: ontology-informed decoding accurately predicts cognitive concepts in unseen tasks. It predicts these concepts better than other commonly used decoders (logistic regression and naive Bayes, see also S6 Text Evaluating prediction accuracy: cross-validation) and NeuroSynth decoding based on meta-analysis. This confirms that the corresponding atlas captures areas specialized in cognitive functions better than conventional approaches.

Very general labels such a “visual” are found in most studies, and therefore easy to predict. However, higher-level or more specialized cognitive concepts such as viewing digits or moving the left foot are seldom present (see S1 Text Distribution of terms in our database). For these rare labels, the fraction of prediction errors is not a useful measure. Indeed, simply assigning them to zero images would lead to a small fraction of errors. For this reason, Fig 5 reports the area under the receiver operating characteristic (ROC) curve. This is a standard metric that summarizes both false positives and false negatives and is not biased for rare labels. This analysis showed that even for relatively rare concepts, successful decoding was possible.

### Regions in our functional atlas

Our approach links different cognitive terms to functionally-specialized brain regions:

#### Visual regions

**Fig 6:**
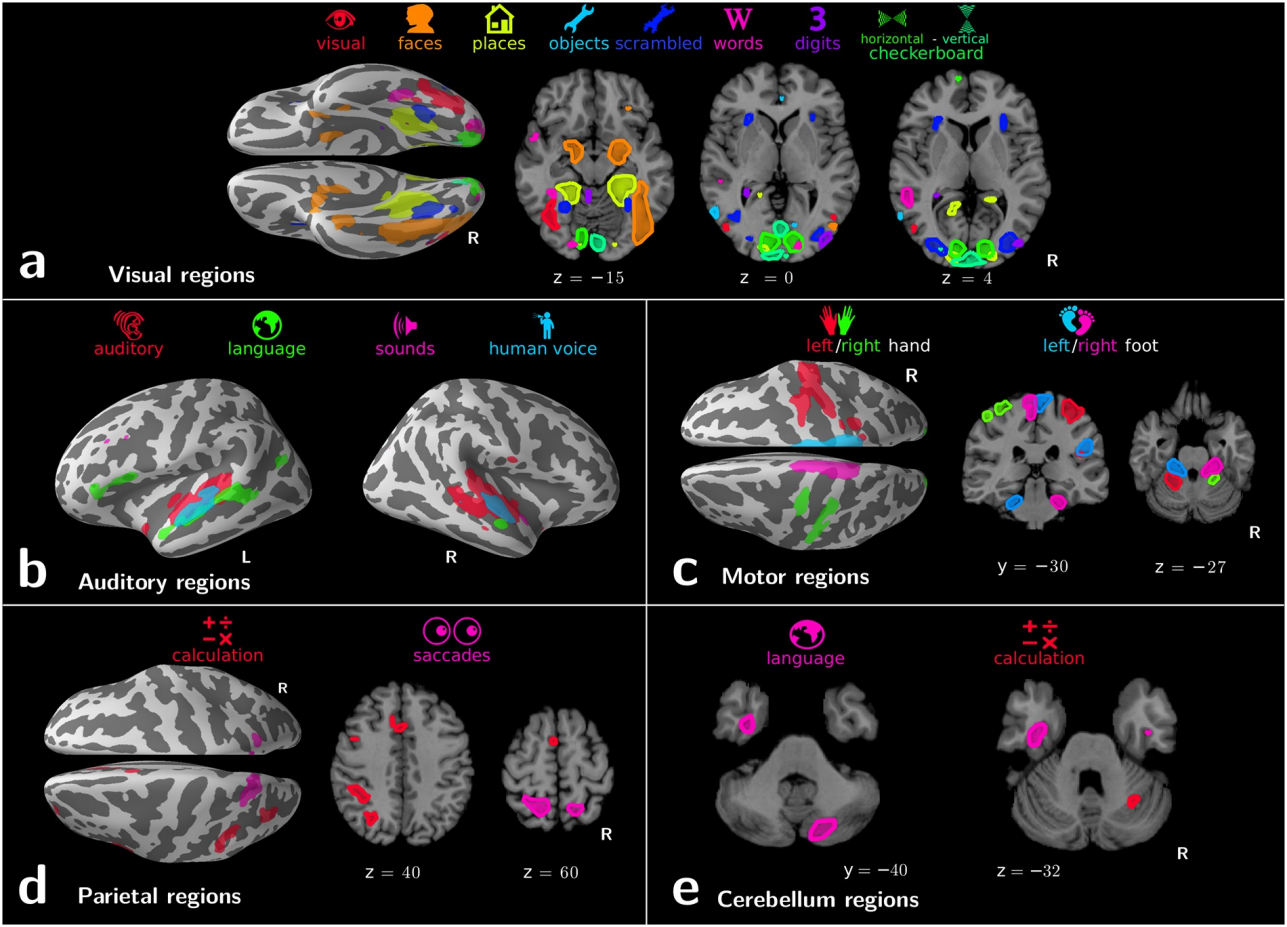
Functional atlases with decoding in an ontology. Regions linked to the various cognitive terms by our mapping approach. They are displayed in 5 different panels depending on their location in the brain: a. visual regions; b. auditory regions; c. motor regions; d. parietal regions; e.cerebellum regions.

(Fig 6a) Visual object recognition is linked to the ventral stream of specialized regions: primary visual areas associated with vertical and horizontal checkerboards in a basic but accurate retinotopic mapping; regions in the LOC linked to objects and scrambled objects; the Fusiform Face Area (FFA) and parahippocampal place area (PPA) associated respectively with “faces” and “places” terms; the region called visual word form area (VWFA) [28] linked to word recognition. Interestingly, both amygdalas also appear related to faces, which could be due to emotional effects of face processing not modeled in the ontology. Digit-viewing does not outline meaningful regions. Corresponding decoding scores are poor (Fig 5): our database is not suited to cross-study mapping of digit viewing. This example confirms that decoding scores can serve as Occam’s razor, validating or falsifying functional regions.

#### Auditory regions

(Fig 6b) Four cognitive terms are represented in the temporal lobe: “auditory”, “sounds”, “language”, and “human voice”. These correspond to increasingly specific concepts in our ontology, and map increasingly focal regions: The “auditory” label denotes the stimulus modality, a fairly general concept, and is linked to the entire auditory cortex. The more precise “sounds” label is associated with Heschl’s gyrus. The “language” label highlights a prototypical left-lateralized language network: anterior and posterior superior temporal sulcus (STS), temporal lobe, supramarginal gyrus, and Broca’s area. The “human voice” label reveals regions in the upper bank of the STS that were previous identified as voice-selective regions by contrasting human voices with closely-matched scrambled voices control conditions [29]. That the mapping singles out such regions from the data is an impressive feat given that only one study in our database [30] features both human voices and non-voice auditory conditions.

#### Motor regions

(Fig 6c) Motor labels reveal the lateralized hands and feet representations in the primary motor cortex, as well as in the cerebellum.

#### Parietal regions

(Fig 6d) Saccadic eye movements and mental arithmetic are known to recruit almost overlapping parietal areas [2], which are difficult to separate with standard analysis. In the IPS (intra-parietal sulcus), we find bilateral regions for saccades but calculation appears left lateralized, consistent with previous reports [31]. Cross-study analysis of activation maps is important to study such nearly-colocalized functions from different cognitive domains. Indeed, meta-analysis based on coordinates suffers a loss of spatial resolution (Fig 3e).

#### Cerebellar regions

While the cerebellum is involved in a variety of mental processes, there are very few systematic mapping results. Previous work [32] studied the somatotopic organization of the cerebellum visible on Fig 6c, with an inverted laterality of functional areas with respect to cortical somatotopy. Other higher-level cognitive functions are represented in the cerebellum with the same inversion. Notably our analysis links the “language” term to a right-lateralized cerebellum region in Crus II (Fig 6e), consistent with language studies [33]. Finally, the “calculation” term is also represented in the right cerebellar cortex, in the superior medial section of the lobule VI. This location has been linked to working memory [34]. It appears here linked to calculation, consistent with the fact that mental arithmetic has a strong working-memory component [35], and our cognitive ontology does not explicitly model working memory.

## Discussion

The inference framework introduced here represents a new approach to developing functional atlases of the human brain. It formally characterizes representations for various cognitive processes that evoke overlapping brain responses, and makes it possible to pool many task-fMRI experiments probing different cognitive domains. Existing meta-analysis approaches face the risk of being unspecific, as demonstrated by our standard analysis results on our database (Figs 3 and 4). Databases of coordinates, such as NeuroSynth, can more easily accumulate data on many different cognitive concepts and support formal reverse inference. This data accumulation is promising, but existing reverse-inference approaches do not suffice to fully remove the overlap in functional regions (Fig 6). Our approach gives more differentiated maps for cognitive concepts by analyzing them in a way that leverages the cognitive ontology. They are also sharper, presumably because they are derived from images rather than coordinates. In a multi-modal framework [17], these maps could be combined with resting-state and anatomical data to provide cognitive resolution to brain parcellations. Note that our framework is meant to be used at the population level and does not address individual brain mapping or decoding.

### Reverse inference mapping

Our analysis framework overcomes the loss in specificity typical of data aggregation. As a result, it enables analyzing jointly more cognitive processes. These richer models can map qualitatively different information. Analyzing more diverse databases of brain functional images can bring together two central brain-mapping questions: *where* is a given cognitive process implemented, and *what* cognitive processes are represented by a given brain structure. Answers to the “what” question have traditionally been provided by invasive studies or neurological lesion reports. Indeed, in a given fMRI study, brain activity results from the task. Concluding on what processes are implied by the observed activity risks merely capturing this task. Decoding across studies can answer this question, by demonstrating the ability to perform accurate inference from brain activity to cognitive function [36].

Reverse-inference maps are essential to functional brain mapping. A key insight comes from the analysis in NeuroSynth [9]: some brain structures are activated in many tasks. Hence, a standard analysis –forward inference– showing such a structure as activated does not provide much information about what function is being engaged. Reverse inference puts the observed brain activity in a wider context by characterizing the behavior that it implies. The analysis performed in NeuroSynth accounts for the multiple tasks that activate a given structure, performing a Bayesian inversion with the so-called *Naive Bayes* model; however, it does not account for other activation foci in the brain that characterize the function. Put differently, our approach departs from the model used by NeuroSynth for reverse inference by what it conditions upon: NeuroSynth’s model asserts functional specialization *conditional to* other terms, while we condition on other brain locations when predicting concept occurrence. This difference should be kept in mind when interpreting differences between the two types of approaches. The Inferior Temporal Gyrus (ITG), for instance, is more active in object-recognition tasks than in other paradigms. However, observing activity in the ITG does not help deciding whether the subject is recognizing faces or other types of objects: the information is in the Fusiform gyrus. An important difference between reverse-inference maps with a Naive Bayes –as in Neurosynth– and using a linear model –as in our approach– is that the Naive Bayes maps do no capture dependencies across voxels. On the opposite, linear models map how brain activity in a voxel relates to behavior *conditionally* on other voxels. Technically, this is the reason why Neurosynth reverse-inference maps related to object recognition overlap in the IT cortex (Fig 3e) while maps produced by our approach separate the representations of the various terms in the ventral mosaic (Fig 3d).

Another, more subtle, benefit of the two-layer model over more classical multi-label approaches is that it combines the decisions of classifiers based on subsets of the data, such as the OvO classifiers, which helps learning relevant local discriminative information.

In sum, our mapping approach provides a different type of brain maps: They quantify how much observing activity in a given brain location, as opposed to other brain locations, informs on whether the subject was engaged in a cognitive operation.

### Generalizing beyond single studies

Brain functional atlases are hard to falsify: is a functional atlas specific to the experimental paradigms employed to build it, or is it more generally characteristic of human brain organization? The success of statistically-grounded reverse inference, which generalizes to new paradigms from unseen studies, suggests that there must be some degree of generality in the present atlas. In demonstrating this generalization, the present work goes beyond previous work that had shown generalization to new subjects under known task conditions [12], but not to unknown protocols. However, it is worth noting that here too we found that it was easier to predict on held-out subjects (from one of the training studies) than on held-out studies (see S6 Text Evaluating prediction accuracy: cross-validation), consistent with a substantial effect of the specific task (see S2 Text Similarities of activations across the database). Despite this, our ontology-enabled approach was able to successfully predict cognitive processes for new tasks. Interestingly, it opens the possibility to perform prospective decoding analyses on novel data, hence makes it easier to grasp the added information of incoming data.

To enable this generalization across paradigms, we characterize each task by the multiple cognitive concepts that it recruits, that are specified in the ontology. Departing from the subtractions often used in brain mapping, our framework relies on quantifying full descriptions of the tasks. In the context of decoding, this approach leads to *multi-label prediction*, predicting multiple terms for an activation map, as opposed to *multi-class prediction*, used in prior works [12, 16], that assigns each new map to a single class. The use of the multi-label approach combined with an ontology capturing the relationships between terms provides a principled way of modeling the multiple components of cognition and thus avoids the need for hand-crafted oppositions that are customarily used in subtraction studies. Defining good ontologies is yet another challenge for the community, but it is not unlikely that brain imaging will become part of that process [36, 37]. Providing a methodological approach founded on an explicit hierarchy of cognitive concepts would allow to test for different cognitive ontologies, and, provided with a comparison metric, select the best ontology according to the available data. Although the present analysis is limited to a relatively small set of cognitive functions, such an approach will be essential as the field attempts to scale such analyses to the breadth of human cognition.

### Conclusion

To build brain functional atlases that map many cognitive processes, we have found that reverse inference and an ontology relating these processes were key ingredients. Indeed, because of the experimental devices used in cognitive neuroimaging, some regions –e.g. attentional or sensory regions– tend to be overly represented in forward inferences. An ontology encodes the related cognitive processes that must be studied together to best establish forward or reverse inferences.

Using a relatively small number of independent task fMRI datasets, our brain-mapping approach reconciles the conundrum of multiple cognitive processes/labels mapping to often overlapping brain regions in activation studies. More data will enable even more fine-grained process-region mappings. In particular higher-level cognitive processes elude the present work, limited by the amount and the diversity of the studies in our database. Indeed, high-level terms form very rare classes in the datasets employed here (see S1 Text Distribution of terms in our database). With increased data sharing in the neuroimaging community [38], there is a growing opportunity to perform this kind of analysis on a much larger scale, ultimately providing a comprehensive atlas of neurocognitive organization. A major challenge to such analyses is the need for detailed task annotation; whereas annotation of task features such as the response effector is relatively straightforward, annotation of complex cognitive processes (e.g., whether a task involves attentional selection or working memory maintenance) is challenging and often contentious. The utility of the ontology in the present work suggests that this effort is worthwhile, and that the increased utilization of ontologies in cognitive neuroscience may be an essential component to solving the problem of how cognitive function is organized in the brain.

## Supporting information

S1 Text Distribution of terms in our database. (PDF)

S2 Text Similarities of activations across the database. (PDF)

S3 Text Forward analysis: Ontology-based design across studies. (PDF)

S4 Text Reverse inference: Decoding with cognitive ontologies. (PDF)

S5 Text Consensus between forward and reverse inference. (PDF)

S6 Text Evaluating prediction accuracy: Cross-validation. (PDF)

S1 Fig Term distribution. The number of times a term appears in our database, per map, subject, or study.
(TIFF)

S2 Fig Terms distribution in studies. Percentage of term occurrence in each study.
(TIFF)

S3 Fig Histograms of the distances between brain activity images. Pairwise distances across all the images of our 30-study database: comparing all images, images sharing a cognitive label, in the same study, or in the same exact contrast.
(TIFF)

S4 Fig Term effect for the “place” term. The “place” term denotes visual place recognition tasks. As such a task involves viewing images, it recruits also the low-level and mid-level visual areas.
(TIFF)

S5 Fig Terms correlations. Correlation matrix between terms across images.
(TIFF)

S6 Fig Ontology contrasts for the “place” term. We contrast the “place” with other visual recognition tasks as defined in S4 Table: recognizing faces, objects, and scrambled images. The contrast is efficient at suppressing low-level visual areas, but does not completely remove mid-level visual areas. Indeed, mid-level features are probably not balanced across studies, as some objects with no background, some full pictures of objects, and some cropped pictures.
(TIFF)

S7 Fig Maps for consensus between forward and reverse. Left: maps for the different inferences on the “place” concept. Right: the overlaid inferences for this concept. The consensus singles out the PPA for the “place” concept.
(TIFF)

S8 Fig Distributions of the z-scores for forward and reverse inference. For the maps related to the *place* concept. **Right**: raw p-values. **Left**: after normalization.
(TIFF)

S9 Fig Prediction scores for different methods. AUC (area under the curve) of the ROC. OD: ontology decoding, LOG: logistic, NB: Naive Bayes. Left: leave-subject-out cross-validation, Right: leave-study-out cross-validation.
(TIFF)

S1 Table Studies in the database. The subject-level maps output by our preprocessing and first-level analysis have been uploaded to NeuroVault, for reproducibility of the analysis. These maps form the input of our analytic scheme. All the subject-level statistical maps that we computed as part of our preprocessing are available on http://neurovault.org/collections/1952.
(XLSX)

S2 Table First-level classifiers used. We train three types of classifier to learn the hierarchy of terms: category classifiers (with a OvA approach), and terms classifiers (both with OvA and OvO approaches). The classifiers’ decision functions span an intermediate feature space tailored to our ontology, upon which we perform a standard OvA approach to predict our labels.
(XLSX)

S3 Table Prediction scores for different methods. AUC (area under the curve) of the ROC curve. OD: ontology decoding, LOG: logistic regression, NB: Naive Bayes, NS: NeuroSynth. The OD (ontology decoding) method performs very well (chance is at .5), including when predicting to new studies. Leave-subject-out cross-validation scheme tend to display a higher prediction score than with a leave-study-out cross-validation. This higher prediction accuracy corroborates the observation that activations in the same study are more similar than activations related to the same cognitive term (S3 Fig).
(TIFF)

S4 Table Terms and categories we use to characterize tasks associated with images in our database. We used CogPO categories for task-related description, and add necessary terms from Cognitive Atlas to describe higher-level cognitive aspect. Here we report only terms that were present in more than one study—aside from the “left foot”, which maps in the analysis as maps in “feet” task category, but not “right foot”. The task categories group terms typically used as conditions and their controls to test a hypothesis. The *stimulus modality* category stands for CogPO and task categories. Some terms do not belong to any task category and are referred as such. The *arithmetics* task category spans across the *response modality* and *instructions* CogPO categories.
(XLSX)
