# Supplementary material for: Atlases of cognition with large-scale human brain mapping
Source: PLoS Comput Biol. 2018 Nov 29;14(11):e1006565. doi: 10.1371/journal.pcbi.1006565 (PMC6289578; doi:10.1371/journal.pcbi.1006565)
Supplement: S1 Text — (PDF) [file pcbi.1006565.s001.pdf]

1 Distribution of terms in our database

Our database is comprised of data from 30 studies, assembled from various sources. We have uploaded the subject-level maps resulting from our first-level analysis on NeuroVault. These form the inputs to our approach. We list in S1 Table references to these datasets as well as the NeuroVault URLs to download the maps.

References

1. Barch DM, Burgess GC, Harms MP, Petersen SE, Schlaggar BL, Corbetta M, et al. Function in the human connectome: task-fMRI and individual differences in behavior. Neuroimage. 2013;80:169–189.
2. Amalric M, Dehaene S. Origins of the brain networks for advanced mathematics in expert mathematicians. Proceedings of the National Academy of Sciences. 2016;113(18):4909–4917.
3. Cauvet E. Traitement des Structures Syntaxiques dans le langage et dans la musique. Paris 6; 2012.
4. Hara N, Cauvet E, Devauchelle A, DEHAENE S, PALLIER C, et al. Neural correlates of constituent structure in Language and Music. Neuroimage. 2009;47:S143.
5. Devauchelle AD, Oppenheim C, Rizzi L, Dehaene S, Pallier C. Sentence syntax and content in the human temporal lobe: an fMRI adaptation study in auditory and visual modalities. Journal of Cognitive Neuroscience. 2009;21(5):1000–1012.
6. Schonberg T, Fox C, Mumford J, Congdon C, Trepel C, Poldrack R. Decreasing ventromedial prefrontal cortex activity during sequential risk-taking: an fMRI investigation of the balloon analog risk task. Frontiers in Neuroscience. 2012;6.
7. Aron A, Gluck M, Poldrack R. Long-term test–retest reliability of functional MRI in a classification learning task. Neuroimage. 2006;29:1000.
8. Xue G, Poldrack RA. Rhyme judgment;. https://openfmri.org/dataset/ds000003.
9. Xue G, Poldrack R. The neural substrates of visual perceptual learning of words: implications for the visual word form area hypothesis. J Cognitive Neurosci. 2007;19:1643.
10. Xue G, Aron A, Poldrack R. Common neural substrates for inhibition of spoken and manual responses. Cerebral Cortex. 2008;18:1923.
11. Aron AR, Behrens TE, Smith S, Frank MJ, Poldrack RA. Triangulating a cognitive control network using diffusion-weighted magnetic resonance imaging (MRI) and functional MRI. The Journal of Neuroscience. 2007;27:3743–3752.
12. Cohen JR. The development and generality of self -control. UCLA; 2009.
13. Foerde K, Knowlton B, Poldrack R. Modulation of competing memory systems by distraction. Proc Natl Acad Sci. 2006;103:11778.
14. Rizk-Jackson A, Aron AR, Poldrack RA. Classification learning and stop-signal (1 year test-retest);. https://openfmri.org/dataset/ds000017.

15. Alvarez RP, Poldrack RA. Cross-language repetition priming;. https://openfmri.org/dataset/ds000051.

16. Alvarez RP, Jasdzewski G, Poldrack RA. Building memories in two languages: An fMRI study of episodic encoding in bilinguals. In: Society for Neuroscience Abstracts; 2002.

17. Poldrack R, Clark J, Pare-Blagoev E, Shohamy D, Creso Moyano J, Myers C, et al. Interactive memory systems in the human brain. Nature. 2001;414:546.

18. Kelly A, Milham M. Cross-language repetition priming;. https://openfmri.org/dataset/ds000101.

19. Kelly A, Uddin LQ, Biswal BB, Castellanos F, Milham M. Competition between functional brain networks mediates behavioral variability. Neuroimage. 2008;39:527.

20. Haxby J, Gobbini I, Furey M, Ishai A, Schouten J, Pietrini P. Distributed and overlapping representations of faces and objects in ventral temporal cortex. Science. 2001;293:2425.

21. Duncan K, Pattamadilok C, Knierim I, Devlin J. Consistency and variability in functional localisers. Neuroimage. 2009;46:1018.

22. Wager TD, Davidson ML, Hughes BL, Lindquist MA, Ochsner KN. Prefrontal-subcortical pathways mediating successful emotion regulation. Neuron. 2008;59:1037.

23. Moran JM, Jolly E, Mitchell JP. Social-cognitive deficits in normal aging. The Journal of Neuroscience. 2012;32:5553–5561.

24. Uncapher MR, Hutchinson JB, Wagner AD. Dissociable effects of top-down and bottom-up attention during episodic encoding. The Journal of Neuroscience. 2011;31:12613–12628.

25. Gorgolewski KJ, Storkey A, Bastin ME, Whittle IR, Wardlaw JM, Pernet CR. A test-retest fMRI dataset for motor, language and spatial attention functions. GigaScience. 2013;2:1.

26. Gauthier B, Eger E, Hesselmann G, Giraud AL, Kleinschmidt A. Temporal tuning properties along the human ventral visual stream. The Journal of Neuroscience. 2012;32:14433–14441.

27. Wakeman DG, Henson RN. A multi-subject, multi-modal human neuroimaging dataset. Scientific data. 2015;2.

28. Knops A, Thirion B, Hubbard EM, Michel V, Dehaene S. Recruitment of an area involved in eye movements during mental arithmetic. Science. 2009;324:1583.

29. Pinel P, Thirion B, Meriaux S, Jobert A, Serres J, Bihan DL, et al. Fast reproducible identification and large-scale databasing of individual functional cognitive networks. BMC neuroscience. 2007;8:91.

30. Pinel P, Dehaene S. Genetic and environmental contributions to brain activation during calculation. NeuroImage. 2013;81:306.

31. Bzdok D, Varoquaux G, Grisel O, Eickenberg M, Poupon C, Thirion B. Formal models of the network co-occurrence underlying mental operations. PLoS Comput Biol. 2016;12:e1004994.

32. Vagharchakian L, Dehaene-Lambertz G, Pallier C, Dehaene S. A temporal bottleneck in the language comprehension network. The Journal of Neuroscience. 2012;32:9089–9102.

33. Salimi-Khorshidi G, Smith SM, Keltner JR, Wager TD, et al. Meta-analysis of neuroimaging data: a comparison of image-based and coordinate-based pooling of studies. Neuroimage. 2009;45:810.

34. Varoquaux G, Gramfort A, Thirion B. Small-sample brain mapping: sparse recovery on spatially correlated designs with randomization and clustering. ICML. 2012;.

35. Dietterich TG. Ensemble methods in machine learning. In: Multiple classifier systems. Springer; 2000. p. 1–15.

36. Hoyos-Idrobo A, Schwartz Y, Varoquaux G, Thirion B. Improving sparse recovery on structured images with bagged clustering. In: Pattern Recognition in NeuroImaging (PRNI), 2015 International Workshop on. IEEE; 2015. p. 73–76.

37. Schwartz Y, Thirion B, Varoquaux G. Mapping paradigm ontologies to and from the brain. Advances in Neural Information Processing Systems. 2013; p. 1673–1681.

38. Breiman L. Stacked regressions. Machine learning. 1996;24:49.

39. Wellcome Department of Cognitive Neurology. SPM8; 2008. http://www.fil.ion.ucl.ac.uk/spm.

40. Pedregosa F, Varoquaux G, Gramfort A, Michel V, Thirion B, Grisel O, et al. Scikit-learn: Machine Learning in Python. Journal of Machine Learning Research. 2011;12:2825.

41. Abraham A, Pedregosa F, Eickenberg M, Gervais P, Mueller A, Kossaifi J, et al. Machine learning for neuroimaging with scikit-learn. Frontiers in Neuroinformatics. 2014;8:14.

42. Haufe S, Meinecke F, Görgen K, Dähne S, Haynes JD, Blankertz B, et al. On the interpretation of weight vectors of linear models in multivariate neuroimaging. Neuroimage. 2014;87:96–110.

43. Poldrack RA, Halchenko YO, Hanson SJ. Decoding the large-scale structure of brain function by classifying mental states across individuals. Psychol Sci. 2009;20:1364.

44. Poldrack RA, Barch D, Mitchell J, Wager T, Wagner A, Devlin J, et al. Towards open sharing of task-based fMRI data: The OpenfMRI project. Front Neuroinform. 2013;7:12.
