# Supplementary material for: Atlases of cognition with large-scale human brain mapping
Source: PLoS Comput Biol. 2018 Nov 29;14(11):e1006565. doi: 10.1371/journal.pcbi.1006565 (PMC6289578; doi:10.1371/journal.pcbi.1006565)
Supplement: S2 Text — (PDF) [file pcbi.1006565.s002.pdf]

## 2 Similarities of activations across the database

To understand how the variance is distributed in our database, we compute the pairwise distances between any two brain activity images in the database. In S3 Fig. we show histograms of distance for all image pairs in the database, then images sharing a cognitive label, images taken from the same study, or corresponding to the same exact experimental contrast. We can see that while images drawn from the same contract or the same study tend to be close (small distance), distances between images sharing a cognitive label are distributed no differently than distances between images drawn randomly in the database.

These distributions show that, despite our uniform preprocessing, the inter-study variance is larger that the variance across labels of cognitive concepts. Indeed, images drawn from the same study tend to be closer than images sharing a cognitive label. Such lack of similarity is evidence that the idiosyncrasies of the experiment –imaging details but also implementation details of the paradigm such as specific choice of stimuli– can explain more variance than the cognitive concepts we are interested in capturing for large-scale functional mapping of the brain.
