# Supplementary material for: Atlases of cognition with large-scale human brain mapping
Source: PLoS Comput Biol. 2018 Nov 29;14(11):e1006565. doi: 10.1371/journal.pcbi.1006565 (PMC6289578; doi:10.1371/journal.pcbi.1006565)
Supplement: S3 Text — (PDF) [file pcbi.1006565.s003.pdf]

3 Forward analysis: ontology-based design across studies

3.1 Modeling brain response to cognitive-ontology concepts

In a standard GLM framework, we use a design matrix capturing the effect on brain activity of the presence of a term in the task description, followed by a set of contrasts to isolate contribution of the term of interest opposed to related terms in the ontology.

Term effect We assign a set of terms to each image, forming a *one-hot-encoding* of the database, *i.e.* representing the occurrence of terms by a binary design matrix. We follow the standard fMRI analysis framework and perform a General Linear Model (GLM). This gives the correlation of each separate voxel with the terms within a set of images, and enables to test for their significance. Using the GLM formulation:

$$\boldsymbol{y} = \boldsymbol{X}\boldsymbol{\beta} + \varepsilon,$$

$\boldsymbol{y}$ corresponds to the activation maps, $\boldsymbol{X}$ to the design matrix modeling the presence of terms, and $\boldsymbol{\beta}$ to the term effects. The input activation maps are subject-level condition versus baseline maps. S4 Fig. shows the effect map for the *places* term. We will use this term in the following to illustrate the differences between the types of inference.

Correlations in the terms induce correlations in the design matrix: effects of terms that appear always together in tasks cannot be teased out. S5 Fig. shows this correlation matrix for our database. We can see that the “visual” and “auditory” terms are very anticorrelated (their correlation is -.9). Indeed, our tasks are exclusively either visual or auditory, aside from the `ds114` study in which there is no explicit stimuli. For this reason, we remove the regressor “auditory”. The auditory map can be defined as the negated map for the visual term. Other terms suffer from strong correlation, in particular the “voice” and “auditory” terms, as most auditory stimuli are voices. However, some tasks involved non-voice auditory stimuli, such as the *muslang* study (see S2 Fig). Using contrasts, as detailed below, can then separate the terms corresponding to multiple different types of auditory stimuli.

Term contrasts A GLM estimates responses for each voxel with respect to a combination of terms. This entails that maps corresponding to the individual term effects show a certain degree of specificity: the effect of that term is *conditional* to the other terms. However, there is shared variance between the terms. To better isolate cognitive processes, a standard analysis in individual studies relies on contrasts in the GLM, e.g., a “face versus place” and a “face versus scrambled picture” contrast for a face recognition study. To disentangle the experimental factors without a too strong a priori on the control conditions, the alternative is to contrast a $\boldsymbol{\beta}$ map against all others, e.g., “face versus place and scrambled picture”. To define such contrasts in a systematic way for the wide array of cognitive concepts touched in our database, we use the categories of our ontology. We form groups of terms within the task categories described in S4 Table: these are used to define the conditions and their controls. Inside each group, we perform a GLM analysis with all the “one versus all” contrasts. We denote these *ontology contrasts*. Note that we do not perform a 3rd level analysis [1] in the sense that the term effects are estimated directly from the subject-level maps, jointly across all studies.

Other regression approaches As outlined by one reviewer another potential approach to drawing relationships between cognitive concepts and brain activity is to rely on Partial Least Squares of Canonical Correlation Analysis methods – or more

precisely, their predictive variants, namely reduced rank regression. These methods typically find combinations of terms that are highly correlated with combination of regional activities. However, they tend to combine many terms to form their prediction, creating latent factors –“loadings”– distributed across labels. In the present work we prefer to rely on term-specific mappings that avoid the additional difficulty of studying the cognitive loadings of the obtained components. The combination across terms is then done explicitly through contrasts and discriminative models.

References

1. Salimi-Khorshidi G, Smith SM, Keltner JR, Wager TD, et al. Meta-analysis of neuroimaging data: a comparison of image-based and coordinate-based pooling of studies. Neuroimage. 2009;45:810.
