# Supplementary material for: Atlases of cognition with large-scale human brain mapping
Source: PLoS Comput Biol. 2018 Nov 29;14(11):e1006565. doi: 10.1371/journal.pcbi.1006565 (PMC6289578; doi:10.1371/journal.pcbi.1006565)
Supplement: S4 Text — (PDF) [file pcbi.1006565.s004.pdf]

4 Reverse inference: Decoding with cognitive ontologies

Linear decoders with good map recovery We want to build a linear model to be able to map the predictive features onto the brain. Feature recovery is the ability to recover stable and meaningful predictive features from our model. Three issues usually get in the way in fMRI multivariate analyses: the high dimensionality of the data, the local correlation of the features –voxels–, and model selection. [1] show that it is possible to come around the dimensionality and correlation problems by using sparse regression models with randomization techniques and feature clustering. This actually amounts to building an ensemble of sparse linear classifiers [2], on a set of randomized parcellations generated by a Ward agglomerative clustering algorithm combined with a resampling method. Note that parcel-averages of the signals are used in the next steps. We add a cross validation procedure in the training of our ensemble in order to select the model. For each random parcellation, we keep the best model. Ensemble classifiers typically either use a voting or an averaging strategy for the final prediction. We choose the latter to keep a linear model, in line with our brain mapping goals. We also perform a non-conservative univariate screening of the features, and keep 30% of the features. This step is primarily due to computational concerns. On the specifics of our model, we choose to use an ℓ_1-logistic regression, and 5K parcels for the clustering. We run 5-fold cross validation for model averaging. For each fold, following [3, 4], we set the ℓ_1 regularization parameter to minimize the error on the left-out data. We average the resulting models.

Class imbalance and rare classes problem The class imbalance problem is inherent to our data since mental processes are not uniformly investigated in the literature, and even less so in our database. This is a common problem for meta-analyses, known as the literature bias. There are several ways to account for class imbalance such as using resampling methods or decomposition strategies to project the classes samples into a balanced space. We choose to use a resampling method akin to bagging (Bootstrap AGGregatING), in which each classifier is given a balanced sub-sample of the whole dataset. This results in an ensemble of classifiers that retains a good coverage of the majority class but suffers less from the imbalanced class distributions.

Hierarchical decoding: using the ontology for an intermediate feature space The previous paragraphs describe the necessary steps to build a classifier for a single label, *i.e.* a single term, but we are in a multi-label classification setting. The usual approach to solve this kind of problem in machine learning is to train one binary classifier per label in a One versus All (OvA) scheme. The approach has successfully been used in our initial contribution [5], but in our opinion suffers from two main limitations in this context. First an OvA classification models each label separately, and by doing so misses potentially useful connections between the labels that could improve their individual prediction. Second, it ignores the experimental design of the studies from which the images are drawn: an OvA approach uses blindly all the data to learn a label, regardless of whether the images are from a study designed to expose this kind of mental process.

We introduce a new model to alleviate these shortcomings, that relies on **stacked regressions** [6]. A stacked regression model is an ensemble method that uses the linear combinations of different classifiers to improve the final prediction. The general idea of this model is to generate different predictors on the same data. The predictors can be generated through resampling methods, or merely use different underlying models (*e.g.*

to combine a collection of linear and non-linear models). We *stack* the decision functions from this *first-level* collection of classifiers, and use them to train a final, *second-level*, predictor that forms a linear combination of the base models. This model has the advantage of building a linear classifier if we avoid introducing non-linearities in the ensemble classifiers. Another interesting property is that it enables to use classifiers that do multi-class prediction, *ie* choose *one* label, to perform multi-label classification, *ie* predict the presence or not of multiple labels. It does so by combining their predictions. Finally, the first level may be seen as a supervised dimensionality reduction method, as we condense the original space to a number of dimensions equal to the number of base classifiers in the ensemble. Note that as all classifiers combined are linear, the resulting complete model is also a linear model, which means that its weights form brain maps.

Software aspects Standard preprocessing was performed with SPM [7]. The ontology-informed decoder as well as the other decoding experiments were implemented using classifiers from scikit-learn [8] with the nilearn toolbox [9] for data preparation steps.

References

1. Varoquaux G, Gramfort A, Thirion B. Small-sample brain mapping: sparse recovery on spatially correlated designs with randomization and clustering. ICML. 2012;.

2. Dietterich TG. Ensemble methods in machine learning. In: Multiple classifier systems. Springer; 2000. p. 1–15.

3. Hoyos-Idrobo A, Schwartz Y, Varoquaux G, Thirion B. Improving sparse recovery on structured images with bagged clustering. In: Pattern Recognition in NeuroImaging (PRNI), 2015 International Workshop on. IEEE; 2015. p. 73–76.

4. Hoyos-Idrobo A, Varoquaux G, Schwartz Y, Thirion B. FReM - Scalable and stable decoding with fast regularized ensemble of models. Neuroimage; 2017 S1053-8119(17)30818-2.

5. Schwartz Y, Thirion B, Varoquaux G. Mapping paradigm ontologies to and from the brain. Advances in Neural Information Processing Systems. 2013; p. 1673–1681.

6. Breiman L. Stacked regressions. Machine learning. 1996;24:49.

7. Wellcome Department of Cognitive Neurology. SPM8; 2008. http://www.fil.ion.ucl.ac.uk/spm.

8. Pedregosa F, Varoquaux G, Gramfort A, Michel V, Thirion B, Grisel O, et al. Scikit-learn: Machine Learning in Python. Journal of Machine Learning Research. 2011;12:2825.

9. Abraham A, Pedregosa F, Eickenberg M, Gervais P, Mueller A, Kossaifi J, et al. Machine learning for neuroimaging with scikit-learn. Frontiers in Neuroinformatics. 2014;8:14.
