# Supplementary material for: Atlases of cognition with large-scale human brain mapping
Source: PLoS Comput Biol. 2018 Nov 29;14(11):e1006565. doi: 10.1371/journal.pcbi.1006565 (PMC6289578; doi:10.1371/journal.pcbi.1006565)
Supplement: S5 Text — (PDF) [file pcbi.1006565.s005.pdf]

5 Consensus between forward and reverse inference

By taking into account several cognitive concepts at the same time, reverse inference maps are more specific than the ones from forward inference, but may also capture irrelevant noise. Indeed, regions that are not marginally[1] linked to the concept, e.g. noise regions, can be included because, conditioning on them removes noise [1]. These regions are not linked to the concept of interest in a forward inference, even with a low threshold. We thus want to use forward inference to remove them from reverse inference, capturing the consensus between the two approaches, as in S7 Fig.

However, using both inferences in conjunction is not straightforward, as they do not perform the same statistical tests and do not have the same statistical power. As we are only interested in the common patterns between both approaches, we use a noise independent procedure to delineate those patterns. Specifically, we compute z-scores for the classifier coefficients by dividing the raw coefficients by their standard error (obtained by cross-validation). The scores' distributions are displayed on the right of S8 Fig., and shows the difficulty to find a scale at which to threshold forward and reverse maps to find the common patterns. For this reason, we normalize independently the forward and reverse maps. The left of S8 Fig. shows the z-scores' distributions after normalization. From this figure, a fair choice of threshold that yields common patterns lies between $z = 1.5$ and $z = 2$. We mask out the reverse inference maps with those from forward inference using a threshold of 1.5 on the normalized statistic.

References

1. Haufe S, Meinecke F, Görgen K, Dähne S, Haynes JD, Blankertz B, et al. On the interpretation of weight vectors of linear models in multivariate neuroimaging. Neuroimage. 2014;87:96–110.

[1]Marginally in the statistical sense: marginal dependence between two variates as opposed to independence conditionally on others.
