# Supplementary material for: Atlases of cognition with large-scale human brain mapping
Source: PLoS Comput Biol. 2018 Nov 29;14(11):e1006565. doi: 10.1371/journal.pcbi.1006565 (PMC6289578; doi:10.1371/journal.pcbi.1006565)
Supplement: S6 Text — (PDF) [file pcbi.1006565.s006.pdf]

6 Evaluating prediction accuracy: cross-validation

6.1 Model evaluation procedures

Cross-validation scheme To evaluate prediction accuracy, we use a randomized leave-3-study out cross-validation scheme. Using cross-study prediction ensures that the representation of the cognitive labels generalizes across paradigms. Failure to do so might result in over fitting the data, and learning studies idiosyncrasies. This is the first time this type of cross validation is used, as previous multi-study decoding experiments [1, 2] relied on a leave-subject out cross-validation. Given the distribution of labels in the database (S2 Fig), a left-out study only represents a fraction of labels. To measure the prediction error better, we leave out 3 studies in the test set. However each fold enables to test only a subset of the terms. We complete 100 iterations of the cross validation to get a good estimate of the classifiers performance even for the minority classes.

We give results for both for our leave-3-study out cross-validation and for a simpler cross-validation scheme in which left out subjects are drawn randomly from the database

Error metric: AUC for ROC We report the area under the curve (AUC) for the ROC (receiver operator characteristic). This metric summarizes the fraction of misses and false detections on the labels when varying the bias on the decision of the classifier: biasing to a large number of predicted labels to minimize the misses, or conversely being conservative and risking false detections. It is a standard metric used in machine learning to evaluate performance for unbalanced problems. Indeed, for rare classes the compromise between misses and false detections is difficult to capture by reporting only the number of errors. This number is not affected by class imbalance, and it can thus be compared across our various labels.

Other classifiers We also provide classification scores for other common decoders not relying on the ontology: a logistic regression and a naive Bayes classifier. The logistic regression is a linear model, very close to the much used linear SVM (that gives similar results and maps, as its mathematical formulation is not very different). The naive Bayes classifier models voxels as independent, and thus leads to univariate estimation of the weights (though multivariate prediction from them).

6.2 Prediction accuracy results

S9 Fig. summarizes results for prediction accuracy. S3 Table gives details for each class.

We find that imposing the ontology structure is beneficial when predicting to new studies, but not when predicting to new subjects from the same studies. This comes from the fact that when predicting in a given study, there always exists in the training dataset an activation map close to that of the new subject. Thus forcing to focus on the difference between labels is counter productive. The classifier equivalent to our ontology aware classifier, but without the ontology, ie a simple logistic regression, performs best.

The Naive Bayes classifier has an overall performance below that of linear models (S3 Table), suggesting that due to its univariate nature, it cannot capture distributed patterns of activity to predict cognitive labels. In other words, different concepts leads to overlapping brain activity patterns. Estimating them in a linear model, that captures the dependence between voxels (leading to partialing out the activation of other voxels for each voxel), is beneficial for prediction.

References

1. Poldrack RA, Halchenko YO, Hanson SJ. Decoding the large-scale structure of brain function by classifying mental states across individuals. Psychol Sci. 2009;20:1364.

2. Poldrack RA, Barch D, Mitchell J, Wager T, Wagner A, Devlin J, et al. Towards open sharing of task-based fMRI data: The OpenfMRI project. Front Neuroinform. 2013;7:12.
