# Supplementary figures and images for: Atlases of cognition with large-scale human brain mapping
Source: PLoS Comput Biol. 2018 Nov 29;14(11):e1006565. doi: 10.1371/journal.pcbi.1006565 (PMC6289578; doi:10.1371/journal.pcbi.1006565)

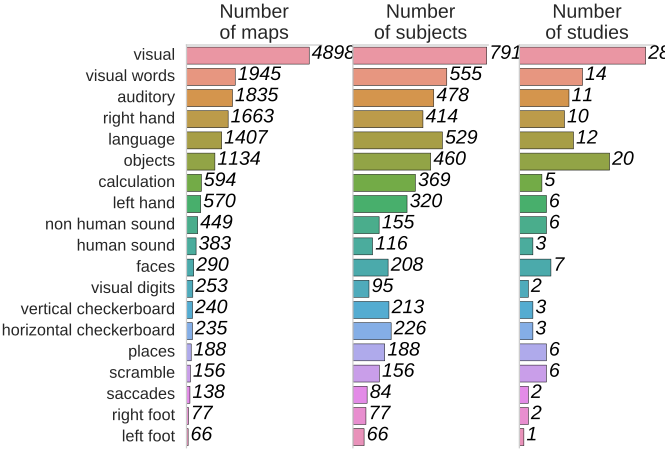

Supplement: S1 Fig — The number of times a term appears in our database, per map, subject, or study. (TIFF) [file pcbi.1006565.s007.tiff]

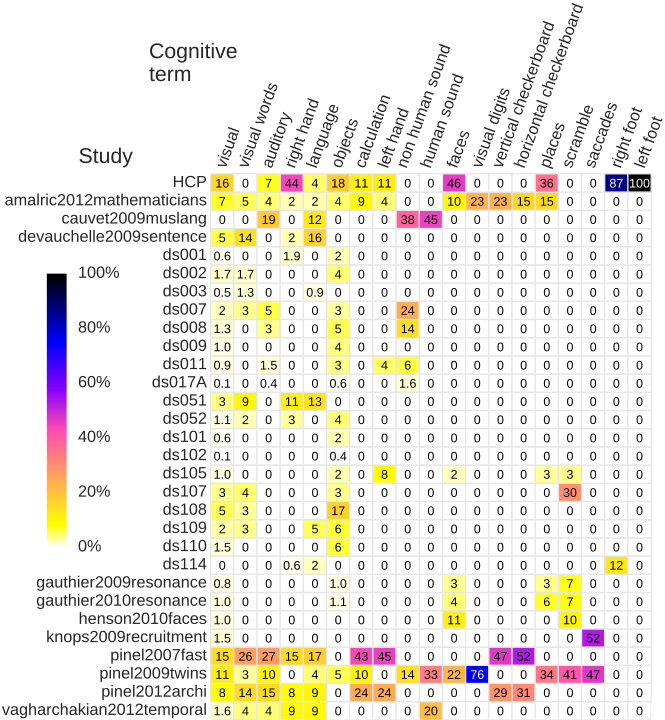

Supplement: S2 Fig — Percentage of term occurrence in each study. (TIFF) [file pcbi.1006565.s008.tiff]

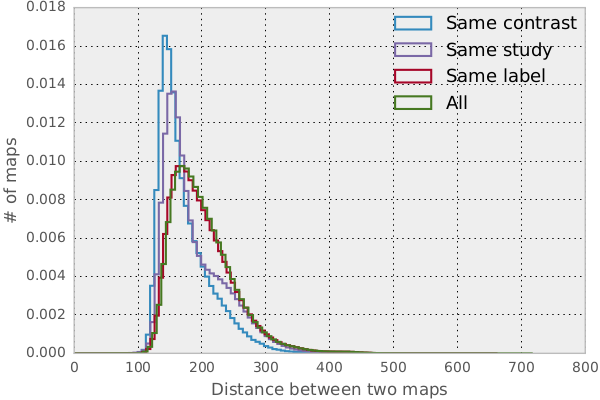

Supplement: S3 Fig — Pairwise distances across all the images of our 30-study database: comparing all images, images sharing a cognitive label, in the same study, or in the same exact contrast. (TIFF) [file pcbi.1006565.s009.tiff]

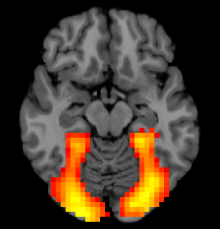

Supplement: S4 Fig — The “place” term denotes visual place recognition tasks. As such a task involves viewing images, it recruits also the low-level and mid-level visual areas. (TIFF) [file pcbi.1006565.s010.tiff]

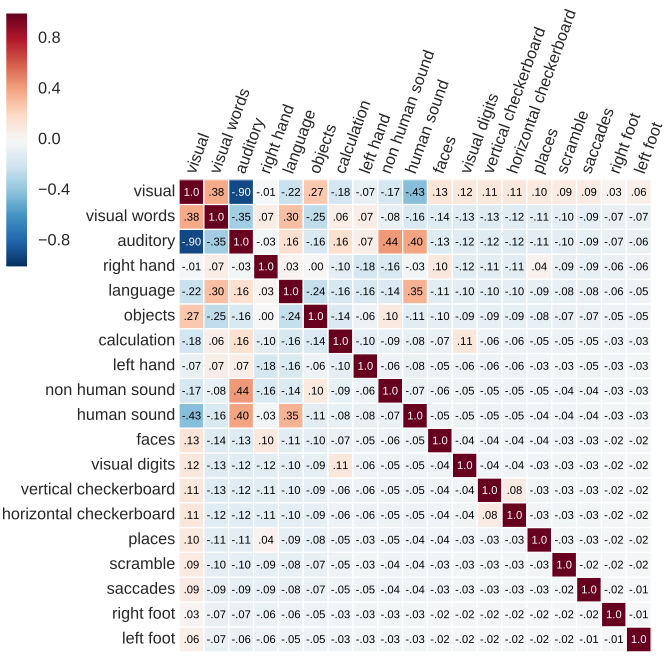

Supplement: S5 Fig — Correlation matrix between terms across images. (TIFF) [file pcbi.1006565.s011.tiff]

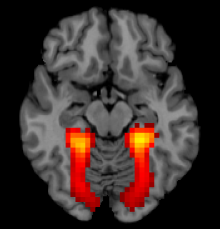

Supplement: S6 Fig — We contrast the “place” with other visual recognition tasks as defined in S4 Table: recognizing faces, objects, and scrambled images. The contrast is efficient at suppressing low-level visual areas, but does not completely remove mid-level visual areas. Indeed, mid-level features are probably not balanced across studies, as some objects with no background, some full pictures of objects, and some cropped pictures. (TIFF) [file pcbi.1006565.s012.tiff]

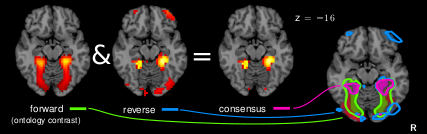

Supplement: S7 Fig — Left: maps for the different inferences on the “place” concept. Right: the overlaid inferences for this concept. The consensus singles out the PPA for the “place” concept. (TIFF) [file pcbi.1006565.s013.tiff]

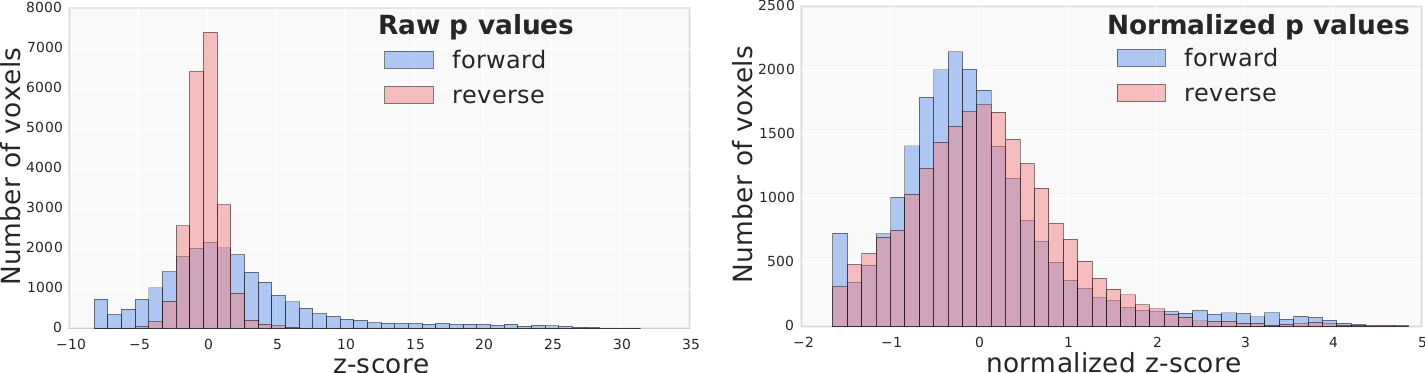

Supplement: S8 Fig — For the maps related to the place concept. Right: raw p-values. Left: after normalization. (TIFF) [file pcbi.1006565.s014.tiff]

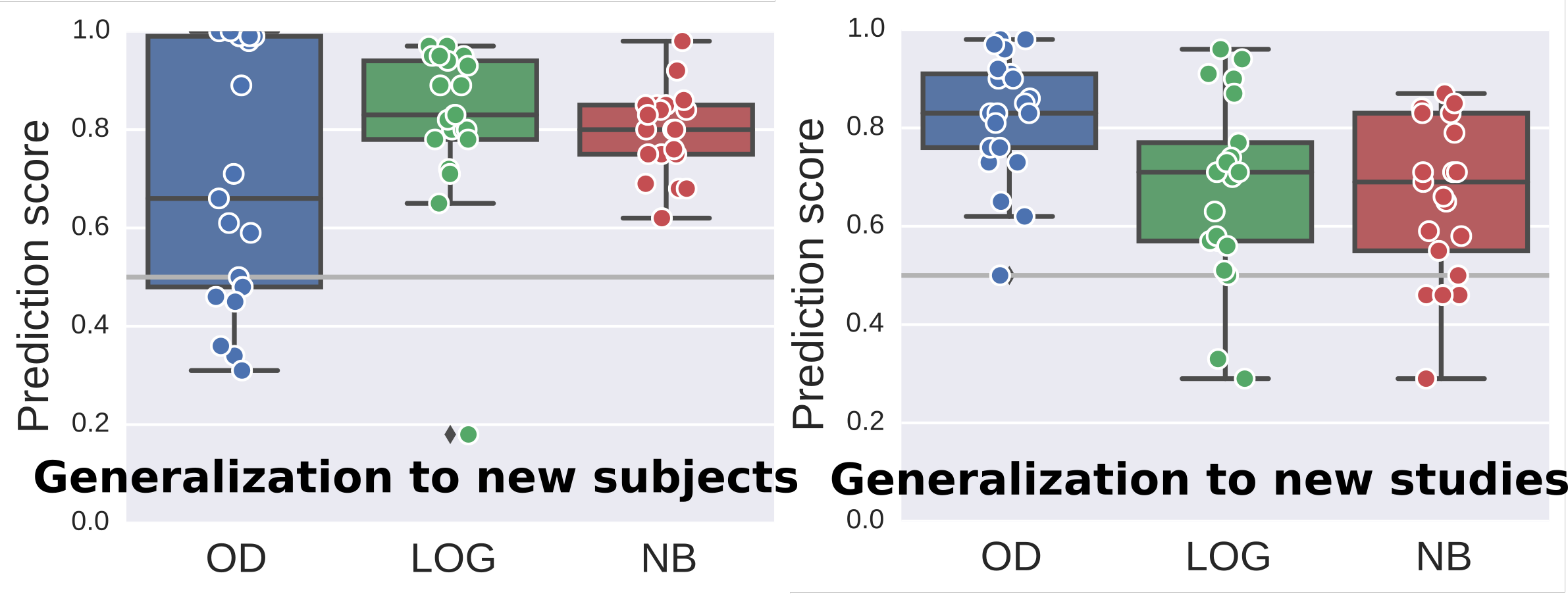

Supplement: S9 Fig — AUC (area under the curve) of the ROC. OD: ontology decoding, LOG: logistic, NB: Naive Bayes. Left: leave-subject-out cross-validation, Right: leave-study-out cross-validation. (TIFF) [file pcbi.1006565.s015.tiff]

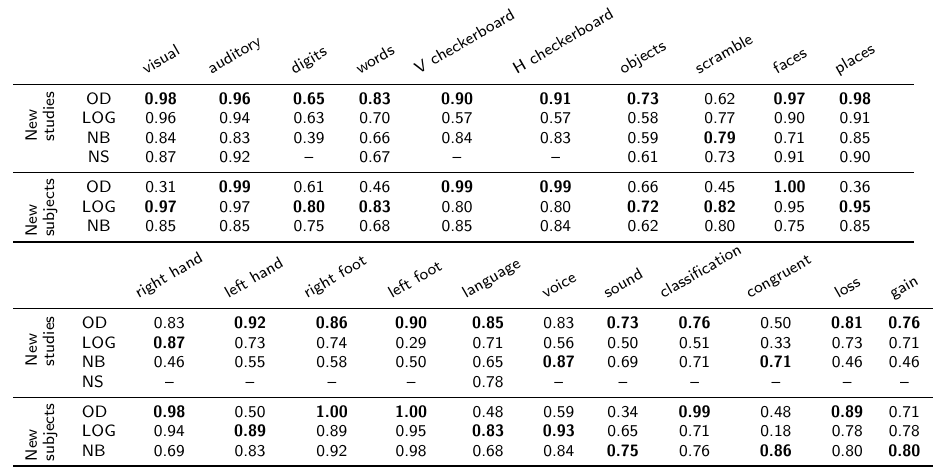

Supplement: S3 Table — AUC (area under the curve) of the ROC curve. OD: ontology decoding, LOG: logistic regression, NB: Naive Bayes, NS: NeuroSynth. The OD (ontology decoding) method performs very well (chance is at .5), including when predicting to new studies. Leave-subject-out cross-validation scheme tend to display a higher prediction score than with a leave-study-out cross-validation. This higher prediction accuracy corroborates the observation that activations in the same study are more similar than activations related to the same cognitive term (S3 Fig). (TIFF) [file pcbi.1006565.s018.tiff]
